# Inhibition of urokinase plasminogen activator with a novel enzyme inhibitor, wxc-340, ameliorates endotoxin and surgery-accelerated growth of murine metastases

**DOI:** 10.1038/sj.bjc.6603550

**Published:** 2007-01-23

**Authors:** S D Killeen, E J Andrews, J H Wang, T Wu, W Schmalix, B Muehlenweg, H P Redmond

**Affiliations:** 1 Department of Surgery, Cork University Hospital, Cork, Ireland; 2 Department of Surgery, University College Cork, Cork, Ireland; 3 Wilex AG, Munich, Germany

**Keywords:** urokinase plasminogen activator (u-PA), metastasis, lipopolysaccaride, WXC-340, surgery

## Abstract

The urokinase plasminogen activator (u-PA) is intimately associated with tumour invasion and metastases. Surgery facilitates accelerated metastatic tumour growth in murine models, a phenomenon related to elevated perioperative bacterial lipopolysaccaride (LPS) and inflammatory cytokine levels. The objectives of the study were to examine the role of u-PA in cytokine-enhanced tumour cell invasion *in vitro* and surgery-induced accelerated metastatic tumour growth *in vivo* and to assess the potential benefit of a novel selective u-PA inhibitor WXC-340 in this setting. CT-26 murine colorectal carcinoma cells were stimulated with LPS, tumour necrosis factor *α* (TNF-*α*) and interleukin 6 (IL-6). Cell supernatant u-PA expression and activity were determined using a colorimetric assay and Western blot analysis, respectively. Baseline and cytokine-stimulated *in vitro* invasion were assessed using ECmatrix invasion chambers. Two established murine models of accelerated metastatic tumour growth were used to investigate the consequences of u-PA inhibition on postoperative metastatic tumour burden. The effect of u-PA inhibition *in vitro* and *in vivo* was examined using the novel selective u-PA inhibitor, WXC-340. Proinflammatory cytokine stimulation significantly enhanced *in vitro* u-PA expression, activity and extracellular matrix invasion by approximately 50% compared to controls (*P*<0.05). This was abrogated by WXC-340. *In vivo* WXC-340 almost completely ameliorated both LPS- and surgery-induced, metastatic tumour growth compared to controls (*P*>0.05). In conclusion, u-PA cascade is actively involved in cytokine-mediated enhanced tumour cell invasion and LPS and surgery-induced metastatic tumour growth. Perioperative u-PA inhibition with WXC-340 may represent a novel therapeutic paradigm.

Surgery remains the only definitive curative modality for colorectal cancer (Meyerhardt and Mayer, 2005). Unfortunately, mounting clinical and experimental evidence suggests that the perioperative milieu may facilitate the progression of occult metastatic disease (DaCosta *et al*, 1998; Mitsudomi *et al*, 1999; Demicheli *et al*, 2001).

Multiple factors have been implicated in this phenomenon including perioperative immune suppression and elimination of putative tumour-suppressing agents with primary tumour excision (Li *et al*, 2001). Surgery produces elevated levels of proinflammatory cytokines such as tumour necrosis factor *α* (TNF-*α*) and interleukin 6 (IL-6), consummate with procedure magnitude (Shiromizu *et al*, 2000). Such proinflammatory cytokines are increasingly implicated in the development and progression of primary and metastatic neoplastic disease (Coussens and Werb, 2002). Furthermore *in vitro* and *in vivo* studies have incriminated perioperative exposure to systemic bacterial endotoxin or lipopolysaccaride (LPS), the primary glycolipid component in the cell wall of Gram-negative organisms, in surgery-induced accelerated metastatic tumour growth, suggesting both a direct cellular role and indirect cytokine-mediated effect. In a murine colorectal carcinoma (CT-26) model of surgery-induced accelerated metastatic tumour growth, laparotomy was associated with a significant elevation in postoperative inflammatory cytokine levels specifically TNF-*α* and IL-6, compared to subjects undergoing laparoscopy or receiving anaesthesia alone (Shiromizu *et al*, 2000). Using the same cell line, Luo *et al* (2004) demonstrated that LPS-mediated enhanced metastatic lung tumour growth was TNF-*α* dependent. In addition, by targeting the effectors mechanisms activated by these proinflammatory cytokines such as the anti-apoptotic cyclooxyenase 2 (COX-2) pathway, it may be possible to modify this accelerated postoperative metastatic tumour growth rate (Qadri *et al*, 2005).

Extracellular matrix invasion is a fundamental pre-requisite for tumour metastases. To facilitate this, cancer cells sabotage native protease systems (Egeblad and Werb, 2002). Indeed, many of the tumour-promoting effects of the aforementioned inflammatory cytokines are secondary to upregulation of proinvasive compounds such as matrix metalloproteases and beta integrins (Wang *et al*, 2003). The urokinase plasminogen activator system comprising urokinase plasminogen activator (u-PA), its receptor urokinase plasminogen activator receptor (u-PAR) (CD87) and two endogenous inhibitors, plasminogen activator inhibitor 1 and 2 (PAI-1, PAI-2) is another such proteolytic system actively subverted by the neoplastic process (Sidenius and Blasi, 2003). Urokinase plasminogen activator catalyses the generation of plasmin from its precursor plasminogen, which in turn promotes the degradation of extracellular matrix components. Binding of u-PA to its receptor facilitates direct plasmin-mediated proteolysis and indirect activation of additional protease systems such as matrix metalloproteases thereby serving to increase the tissue-remodelling ability and invasion capacity of carcinoma cells (Sidenius and Blasi, 2003). Reflecting its clinical relevance, elevated serum and tumour levels of u-PA, u-PAR and PAI-1 are poor prognostic indicators in a variety of neoplasms including colorectal, breast, lung and renal carcinoma (Duffy, 2002). Moreover, proinflammatory cytokines increase the concentration and activity of u-PA produced by human colorectal cancer cells *in vitro* at concentrations similar to postoperative serum levels (Tran-Thang *et al*, 1996).

Thus, the u-PA system is an attractive therapeutic target. Mimetics of the growth factor-like domain of u-PA inhibit experimental and spontaneous metastases in a murine model of lung cancer (Kobayashi *et al*, 1996) and u-PAR antagonists inhibit primary tumour growth and angiogenesis in syngenic mouse models (Min *et al*, 1996). A recently developed selective u-PA inhibitor, WX-UK1 impaired development of lung metastases in a rat model of spontaneous metastatic orthotopic breast adenocarcinoma, whereas Ertongur *et al* (2004) demonstrated that this compound inhibited *in vitro* tumour cell Matrigel invasion by a variety of human cancer cell lines (Setyono-Han *et al*, 2005). WXC-340 is a novel, related second-generation synthetic, low molecular weight protease inhibitor that selectively inhibits activated u-PA in a dose-dependent manner.

Given the prominence of the u-PA system in promoting tumour cell invasion and the upregulation of components of the u-PA system by elevated inflammatory cytokines at concentrations similar to levels seen post laparotomy, inhibition of the u-PA protease system may represent an innovative and potent perioperative therapeutic strategy to attenuate surgery-induced accelerated tumour growth. The aims of this study therefore, were to determine if proinflammatory cytokines at levels similar to those seen postoperatively upregulated u-PA expression and to establish if this increase enhanced u-PA-mediated invasion *in vitro*. We additionally investigated the role of the u-PA system in LPS and surgery-induced accelerated metastatic tumour growth *in vivo* and also ascertained if the novel synthetic u-PA inhibitor, WXC-340 ameliorated *in vitro* cytokine-enhanced tumour cell invasion and *in vivo* surgery and LPS-induced accelerated metastatic tumour growth.

## METHODS

### Cell culture

The murine CT-26 colorectal carcinoma cell line was grown in RPMI 1640 medium containing 10% fetal calf serum, 100 units ml^−1^ penicillin, streptomycin sulphate (100 *μ*g ml^−1^) and 2.0 mmol l^−1^ glutamine. Cells were maintained at 37°C in a humidified 5% CO_2_ atmosphere and subcultured by trypsinisation with 0.05% trypsin–0.02% ethylenediaminetetraacetic acid (EDTA) when confluence approached (all from Life Technologies, Paisley, Scotland).

### Cell stimulation and sample preparation

Cells cultured in six-well plates (Falcon; 1 × 10^6^ cells well^−1^) were exposed to various concentrations of LPS (*Escherichia coli* O55B5) (100, 1000 and 10 000 ng ml^−1^), TNF-*α* (1, 2.5 and 5 ng ml^−1^) and IL-6 (1, 2.5 and 5 ng ml^−1^) for different time periods (0, 6, 12, 18 and 24 h) at 37°C in a humidified 5% CO_2_ environment (all Sigma-Aldrich, St Louis, MO, USA). Conditioned medium was removed, centrifuged at 5000 r.p.m. for 5 min and frozen at −80°C or analysed immediately. Urokinase plasminogen activator blockade involved preincubation with 0.3 *μ*g ml^−1^ WXC-340 (optimal inhibitory concentration was determined from dose–response curves) for 1 h. WXC-340 was kindly provided by Dr Bernd Muehlenweg (Wilex AG, Munich, Germany). Urokinase plasminogen activator activity was also impaired by incubating-cells with 37.5 *μ*g ml^−1^ of a u-PA function-blocking antibody (American Diagnostica, Greenwich, London).

For Western blot analysis of activated u-PA in conditioned culture medium, medium was concentrated 40-fold using centricon 10 ultrafiltration tubes (Millipore, Ireland).

### UPA activity assay

Active murine u-PA levels were quantified by use of a u-PA activity assay kit (Innovative Research, Michigan, USA), in accordance with the manufacturer's instructions.

### The u-PA inhibitor WXC-340

WXC-340 is a synthetic low molecular weight, serine protease, u-PA inhibitor. For this study u-PA activity was initially assessed after incubation with various concentrations of WXC-340 from 0.01 to 1 *μ*g ml^−1^ WXC 340 as shown in Table 1. At 0.3 *μ*g ml^−1^ WXC-340, there was little change in u-PA activity. At concentrations above 1 *μ*g ml^−1^, WXC-340 there was decreased cell viability as assessed by MTT assay. Hence for the stimulation experiments and chemoinvasion assay 0.3 *μ*g ml^−1^ WXC-340 was used.

### Western blot analysis

Equal amounts of protein were resolved on SDS–PAGE, transfer to nitrocellulose membrane, and incubated with specific anti-u-PA and anti-u-PAR antibodies (Santa Cruz, CA, USA) for 18 h at 4°C. After washing and incubating with secondary antibodies, immunoreactive proteins were visualised by the ECL detection system (Amersham Biosciences Piscataway, NJ, USA).

### Tumour invasion assay

*In vitro* tumour cell invasion was assessed using the extracellular matrix (ECmatrix) invasion chamber (Chemicon, Temecula, CA, USA). This consists of a invasion chamber with cell culture inserts containing an 8-*μ*m pore size positron emission tomography membrane lined by a thin layer of ECmatrix (ExtraCellualr matrix, a reconstituted basement membrane matrix of proteins derived from the Engelbreth Holm-Swarm (EHS) mouse tumour), which sits in a 96-well plate that acts as a reservoir for chemoattractant. Briefly, 0.5 ml serum-free medium containing 1 × 10^5^ cells ml^−1^ was added to the cell culture insert of the invasion chamber. This was supplemented with TNF-*α*, Il-6 (2.5 ng ml^−1^) or serum free medium. FBS (20 *μ*g ml^−1^) was added in the outer chamber as a chemoattractant. WXC-340 was added to the upper chamber to assess the effect of u-PA inhibition on *in vitro* invasion. The cells were then incubated at 37°C in humidified 5% CO_2_ conditions for 18 h. Medium in the upper chamber was discarded and the chamber washed. Invaded cells attached to the bottom of the matrix membrane were detached and lysed in cell lysate buffer. Cell lysate was then stained with CyQuant GR Dye (Chemicon, Temecula, CA, USA). Fluorescence was measured using a fluorescence plate reader at an excitation wavelength of 485 nm and an emission wavelength of 520 nm. A standard curve to convert measured fluorescence to cell number was constructed utilising known cell numbers. Values are expressed as the number of invaded cells per 1 × 10^6^.

### Animals

Six- to eight-week-old female Balb/c mice were used in all experiments. Mice were housed in barrier cages under controlled environmental conditions (12/12 h of light/dark cycle, 55±5% humidity, 23°C) and had free access to standard laboratory chow and water. All animal procedures were conducted in the University Biological Services Unit under a license from the Department of Health and Children (Republic of Ireland). Age- and weight-matched mice were used throughout.

### Perioperative proinflammatory cytokine levels

Mice were separated into three groups receiving anaesthesia alone, anaesthesia and intraperitoneal (i.p.) LPS, and anaesthesia and laparotomy, respectively. Three mice per group were killed at each time point 0, 3, 6 and 12 h after surgery (Shiromizu *et al*, 2000) and blood samples were obtained by cardiac puncture. Following centrifugation, serum levels of IL-6 and TNF-*α* were determined by ELISA in accordance with the manufacturer's instructions.

### Experimental CT-26 lung metastatic model and interventions

**Figure 1:**
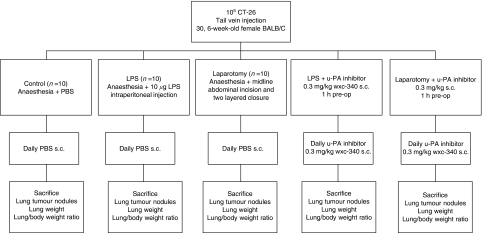
Schematic diagram outlining experimental procedure: 9 days after tail vein injection, animals were anaesthesied and randomised to one of five groups. Group 1 received an i.p. injection of PBS (1 ml), group 2 10 mg LPS (in 1 ml PBS), group 3 underwent laparotomy as described previously, group 4 received 0.3 mg kg^−1^ WXC-340 (in 1 ml PBS) 1 h before intraperitoneal injection of 10 mg ml^−1^ LPS and group 5 received 0.3 mg Kg^−1^ WXC-340 (in 1 ml PBS) 1 h before laparotomy. Subsequently groups 1–3 received subcutaneous PBS (1 ml) daily, whereas groups 4 and 5 received subcutaneous 0.3 mg kg^−1^ WXC-340 (in 1 ml PBS).

Subconfluent tumour cells were harvested and passed through a 40 *μ*m cell strainer, washed twice in PBS and resuspended in PBS at 2 × 10^5^ ml^−1^. Single-cell suspensions of greater than 90% viability based on Trypan Blue exclusion were used. Mice were injected with 10^5^ CT 26 cells via the tail vein. Nine days later, mice were randomised to one of five experimental groups (*n*=50, 10 per group). All animals were anaesthetised with halothane. Group 1 received PBS (1 ml) and group 2, 10 *μ*g LPS (in 1 ml PBS) via i.p. injection as reported by Luo *et al* (2004). Group 3 underwent laparotomy as described previously by Condon *et al* (2004). This group underwent a midline xiphoid to pubis incision, which exposed the peritoneal contents for 15 min before closure (5 min) with a continuous 3/0 nylon suture (Ethicon, Somerville, NJ, USA). These control groups received subcutaneous PBS daily post intervention. Group 4 received subcutaneous WXC-340 1 h before LPS administration, whereas group 5 received subcutaneous WXC-340 1 h before laparotomy. Both these groups received daily subcutaneous WXC-340 thereafter. After 7 days, mice were killed and weighed (Figure 1). The lungs were resected, weighed and lung tumour nodules were counted. Specimens were paraffin-embedded following fixation with 4% formaldehyde in phosphate-buffered saline. Tissue sections, 7 *μ*m thick, were stained with haematoxylin and eosin.

To examine u-PA expression following LPS administration or surgery, mice (*n*=3 per group) were killed 24 h postintervention. Tumour nodules were microdissected and underwent sonication. Urokinase plasminogen activator activity was assessed as described previously. Microdissected tumour cell homogenates were generated by lysis in ice-cold lysis buffer (50 mM Tris–HCl, pH 7.5, 150 mM NaCl, 1% nonident P-40, 1 mM EDTA, 1 mM NaF, 1 mM sodium orthovanadate with freshly added 1 mM phenylmethylsulphonyl fluoride, 0.5 mM DTT, 0.5 mM PMSF and protease inhibitor cocktail (Roche, Mannheim, Germany)), clarified by centrifugation at 7500 g and protein concentrations were determined using a Micro BCA protein assay reagent kit (Pierce, Rockford, IL, USA).

### Statistical analysis

Results are presented as the mean±s.d. Statistical analysis was performed using analysis of variance (ANOVA). Statistical significance was accepted at a *P*-value less than 0.05.

## RESULTS

### Stimulation of CT-26 cancer cells with TNF-*α* and IL-6 leads to enhanced u-PA activity in stimulated cell supernatants that are abrogated by the u-PA inhibitor WXC-340

**Figure 2:**
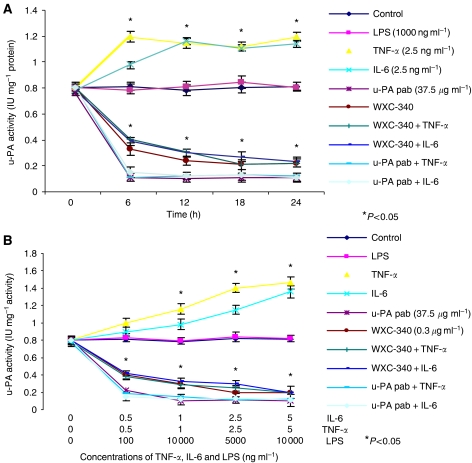
Inflammatory cytokines enhance u-PA activity in a time (**A**) and dose (**B**)-dependent manner. Pretreatment with WXC-340 impaired baseline and cytokine-enhanced u-PA activity. Results are expressed as the mean±s.d. and are representative of four separate experiments, conducted in triplicate. Statistical significance was compared with control cells incubated with culture medium alone (^*^P<0.05).

The urokinase plasminogen activator activity in CT-26 cell supernatant was increased with proinflammatory cytokine (TNF-*α* and IL-6) but not LPS stimulation in a time and dose-dependent manner (Figure 2). Following dose optimisation experiments, both base line and stimulated u-PA activity were abrogated almost completely by pre treatment of cells with WXC-340 at concentrations of 0.3 *μ*g ml^−1^ (*P*=0.039 compared to control groups). Lipopolysaccaride stimulation had no direct effect on u-PA activity in CT-26 cell supernatant.

### Stimulation of CT-26 cancer cells with TNF-*α* and IL-6 leads to enhanced u-PA expression in stimulated cell supernatants

**Figure 3:**
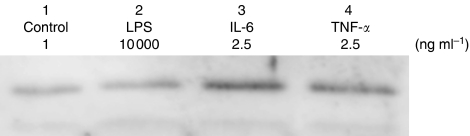
Western blot analysis of concentrated conditioned medium demonstrating increased activated u-PA with cytokine stimulation. (lane 1:control, lane 2: 10 000 ng ml^−1^ LPS, lane 3: 2.5 ng ml^−1^ TNF-*α*, lane 4: 2.5 ng ml^−1^ IL-6).

Mirroring this increased u-PA activity, u-PA expression in concentrated CT-26 supernatant as demonstrated by Western blot analysis was enhanced by stimulation with the proinflammatory cytokines TNF-*α* and IL-6 (Figure 3).

## THE U-PA INHIBITOR WXC-340 AMELIORATES BASELINE AND CYTOKINE STIMULATED *IN VITRO* INVASION BY CT-26 CELLS

**Figure 4:**
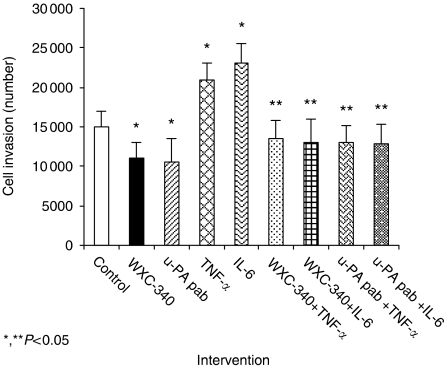
Cytokine stimulation with TNF-α and IL-6 (2.5 ng ml^−1^) significantly enhances CT-26 *in vitro* ECmatrix invasion. This cytokine-mediated increased invasion is inhibited by the novel u-PA inhibitor WXC-340 at a concentration of 0.3 mg ml^−1^. Results are expressed as the mean±s.d. and are representative of four separate experiments, conducted in triplicate. Statistical significance was compared with control cells incubated with culture medium alone (^*^P<0.05) or cytokine stimulated cells (^**^P<0.05).

TNF-*α* and IL-6 accentuated *in vitro* ECmatrix invasion (Figure 4). Baseline CT-26 matrix invasion was significantly inhibited by incubation with WXC-340 (*P*=0.045 compared to control groups). Pre-treatment of CT-26 cells with 0.3 *μ*g ml^−1^ WXC-340 before cytokine stimulation ameliorated cytokine-induced increases in CT-26 invasion *in vitro* (*P*=0.027 compared to control groups) (Figure 4).

### Intraperitoneal LPS and surgery increase proinflammatory cytokine levels

**Figure 5:**
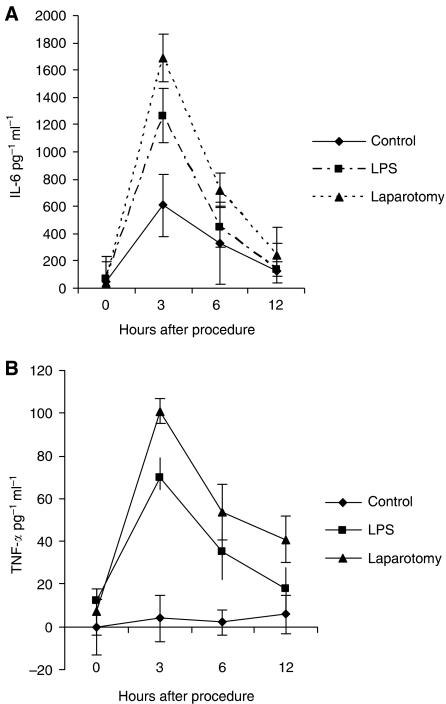
Serum proinflammatory cytokine levels were significantly elevated 3 and 6 h after LPS or laparotomy compared to controls. (^*^P<0.05), *n*=3 mice per group at each time point.

Serum IL-6 and TNF-*α* levels were significantly elevated after laparotomy and ip LPS administration when compared to control groups (Figure 5).

### Intraperitoneal LPS and surgery increase metastatic tumour burden, u-PA expression and activity in microdissected lung metastatic tumour nodules

**Figure 6:**
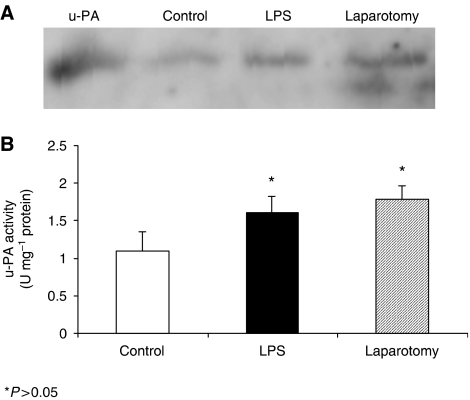
Western blot analysis of microdissected tumour nodules revealed accentuated activated u-PA expression in animals, which had received LPS and undergone surgery compared to control mice (**A**). There was increased u-PA activity in microdissected tumour nodule lysates from in animals which had received LPS and undergone surgery compared to control mice (^*^P<0.05) (**B**). *n*=3 mice per group.

As reported previously both LPS and a standardised laparotomy significantly increased the total metastatic nodule numbers, lung weight and lung weight- to- body ratio compared to animals receiving i.p. PBS (*P*=0.01) (Condon *et al*, 2004). Microdissected tumour nodule lysates from animals in the LPS and surgery groups (*n*=3) demonstrated enhanced u-PA expression on Western blot analysis and u-PA activity on colormetric assay compared to control animals (*P*=0.031) (Figure 6a and b, respectively).

### Abrogation of LPS and surgery stimulated enhanced metastatic tumour burden by the u-PA inhibitor WXC-340

**Figure 7:**
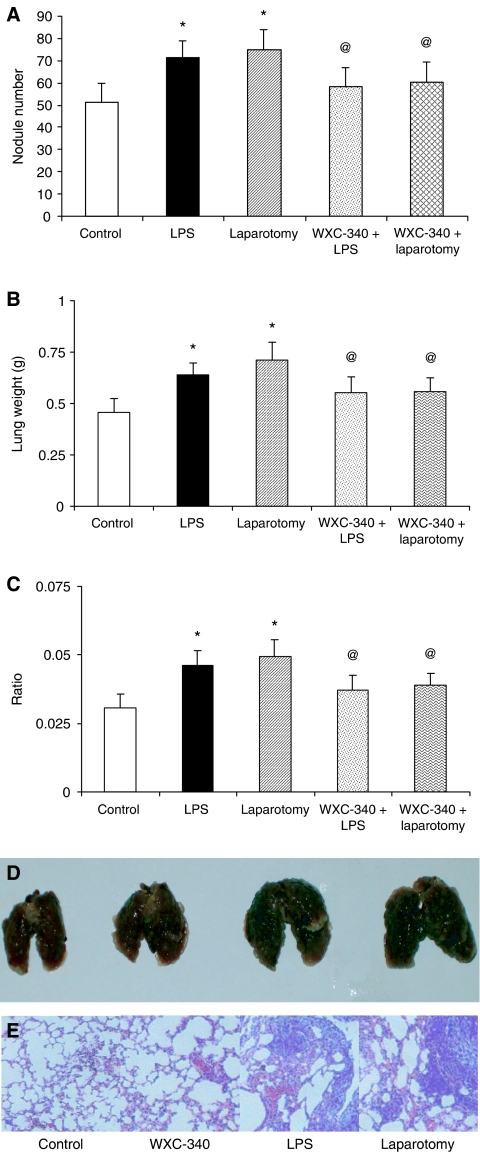
Treatment of animals with WXC-340 abrogated LPS and surgery associated accelerated metastatic tumour growth (^*^P<0.05). Mice that received WXC-340 before LPS administration and subsequently daily subcutaneous WXC-340 (0.3 mg kg^−1^) had significantly lower numbers of tumour nodules (**A**), lung weight (**B**) and lung-to-body weight ratio (**C**) than control animals receiving daily PBS (^@^; *P*<0.05). Gross specimens (**D**) and H and E staining (**E**) demonstrating significantly lower numbers of tumour nodules in the WXC-340-treated mice.

Animals treated with 0.3 mg kg^−1^, WXC-340 (before i.p. LPS or standardised laparotomy, in conjunction with subsequent daily subcutaneous injection) developed fewer lung tumour nodules (Figure 7a), had lower lung weights (Figure 7b) and lung to body weight ratios (Figure 7c) compared to age- and gender- matched controls (*P*<0.019, compared to PBS controls).

### Toxicity profile

Serine protease inhibitors are important components in the coagulation cascade (Setyono-Han *et al*, 2005). There was no evidence of haemorrhagic complications and the coagulation parameters of treated mice were not disturbed. Furthermore, in animals that received WXC-340, all wounds remained intact with no evidence of breakdown. Repeated WXC-340 treatment of mice was well tolerated with no signs of overt toxicity. WXC-340 did not result in changes in body weight or other organs. There was no evidence of skin irritation at the injection site.

## DISCUSSION

Increasing evidence suggests that surgery may accelerate the growth of minimal residual disease. Multiple factors have been implicated in this phenomenon, the plethora of proinflammatory cytokines, specifically TNF-*α* and IL-6, released by the surgical insult, probably play a pre-eminent role (Shiromizu *et al*, 2000; Luo *et al*, 2004). Identification of downstream effector mechanisms utilised by tumour cells to enhance invasion in this setting could highlight novel targets for possible use in this perioperative period of therapeutic opportunity (Qadri *et al*, 2005).

One such effector mechanism subverted by the neoplastic process is the urokinase plasminogen activator system, which directly and indirectly promotes tumour cell invasion and metastasis through multiple mechanisms including plasmin-mediated pericellular proteolysis, enhanced vitronectin adhesion and activation of pro-metastatic intracellular pathways such as src and ERF 1 and 2 (Ahmed *et al*, 2003; Sidenius and Blasi, 2003). Furthermore u-PA is potently upregulated by the proinflammatory cytokines TNF-*α* and IL-6 in a variety of human cancer cell lines *in vitro* and the u-PA system is significantly activated by infection and sepsis *in vivo* as evidenced by increased circulating levels of u-PA (Robbie *et al*, 2000; Abraham *et al*, 2003).

We demonstrate that TNF-*α* and Il-6, the primary inflammatory cytokines elevated by i.p. LPS injection or standardised laparotomy, enhance u-PA secretion and activity by CT-26 murine colorectal carcinoma *in vitro*. This cytokine-mediated enhanced u-PA production is parlayed into increased *in vitro* ECmatrix invasion. The novel u-PA inhibitor WXC-340 inhibits both basal and cytokine augmented *in vitro* invasion, indicative of the prominent role of u-PA in cytokine-enhanced *in vitro* invasion. Using two well-defined models of accelerated metastatic tumor growth, we confirmed that both LPS and standardised laparotomy significantly accentuated metastatic tumour burden. Western blot analysis of micro-dissected metastatic tumor nodules revealed increased u-PA expression in the LPS and surgery groups compared to controls (*n*=3). Furthermore, treatment with subcutaneous WXC-340 ameliorated both the LPS and surgery-induced increases in metastatic tumour load. Collectively these results support a role for u-PA in postoperative tumour growth and may represent a novel therapeutic target in the perioperative setting.

Proinflammatory cytokines, elevated postoperatively, have an emerging role in surgery-induced accelerated growth of metastatic lesions. Although not the primary focus of this study, the findings are in agreement with Luo *et al* (2004) and support the rationale of anti-cytokine interventions in the postoperative phase. WXC-340 is a potent inhibitor of u-PA leading to impairment of the urokinase plasminogen activator cascade and ultimately reduced cancer cell invasion and metastases. Our results suggest that decreased u-PA-dependent proteolysis is the primary pathway involved; however, additional u-PA-mediated mechanisms such as angiogenesis and cell signalling may also be beneficially altered. Urokinase plasminogen activator is a contributor to the coagulation cascade. In our study, there was no evidence of any haemorrhagic complications or derangement of coagulation parameters. Furthermore, there was no skin irritation at the injection site unlike reports involving other u-PA inhibitors (Kobayashi *et al*, 1996).

Other components of the u-PA system, namely u-PAR and PAI-1, promote tumour progression (Duffy, 2002; Sidenius and Blasi, 2003). Expression of u-PAR and PAI-1 by stromal and cellular tumour components are substantially increased by proinflammatory cytokines *in vitro* and sepsis significantly increases serum PAI-1 levels (Robbie *et al*, 2000; Abraham *et al*, 2003). Thus, although this study focused on u-PA and its inhibition, other elements of this cascade are also possibly involved and warrant further investigation. Development of selective synthetic inhibitors of the aforementioned molecules and deployment in the perioperative period signifies an original therapeutic modality.

In conclusion, proinflammatory cytokines increase u-PA expression and activity *in vitro* resulting in enhanced u-PA-mediated extracellular matrix invasion, which is partially ameliorated by u-PA inhibition using the novel agent WXC-340. In two models of surgery-induced accelerated metastatic tumour growth, WXC-340 significantly reduced metastatic burden suggesting that the u-PA cascade is a valid target in the perioperative period.

## Figures and Tables

**Table 1:** u-PA activity after incubation with various concentrations of WXC-340

WXC-340 *μ*g/ml	0.00	0.01	0.05	0.075	0.10	0.20	0.30	0.40	0.50	0.75	1.00	1.50
u-PA activity	0.80 (±0.05)	0.78 (±0.061)	0.61 (±0.037)	0.43 (±0.052)	0.31 (±0.072)	0.23 (±0.046)	0.2 (±0.067)	0.18 (±0.064)	0.16 (±0.051)	0.2 (±0.081)	0.16 (±0.09)	0.15 (±0.087)
